# Does Arch Stiffness Influence Running Spatiotemporal Parameters? An Analysis of the Relationship between Influencing Factors on Running Performance

**DOI:** 10.3390/ijerph18052437

**Published:** 2021-03-02

**Authors:** Felipe García-Pinillos, Diego Jaén-Carrillo, Pedro Ángel Latorre-Román, Carles Escalona-Marfil, Víctor M. Soto-Hermoso, Carlos Lago-Fuentes, Silvia Pueyo-Villa, Irma Domínguez-Azpíroz, Luis E. Roche-Seruendo

**Affiliations:** 1 Department of Physical Education and Sports, University of Granada, 18010 Granada, Spain; vsoto@ugr.es; 2 Department of Physical Education, Sports and Recreation, Universidad de La Frontera, 1145 Temuco, Chile; 3 Campus Universitario, Universidad San Jorge, 50830 Villanueva de Gállego Zaragoza, Spain; djaen@usj.es (D.J.-C.); leroche@usj.es (L.E.R.-S.); 4 Department of Corporal Expression, University of Jaen, 23071 Jaén, Spain; platorre@ujaen.es; 5 Faculty of Health Sciences, Universidad Central de Cataluña, 08500 Barcelona, Spain; cescalona@umanresa.cat; 6 Sport and Health University Research Institute (iMUDS), 18007 Granada, Spain; 7 Department of Education, Universidad Internacional Iberoamericana, 24560 Campeche, Mexico; carlos.lago@uneatlantico.es (C.L.-F.); irma.dominguez@uneatlantico.es (I.D.-A.); 8 Faculty of Health Sciences, Universidad Europea del Atlántico, 39011 Santander, Spain; 9 Department of Languages and Education, Universidad Europea del Atlántico, 39011 Santander, Spain; silvia.pueyo@uneatlantico.es; 10 Research Group on Foods, Nutritional Biochemistry and Health, Universidad Europea del Atlántico, 39011 Santander, Spain

**Keywords:** arch height, arch mobility, endurance runners, foot, spring-mass model

## Abstract

This study aimed to determine the influence of arch stiffness on running spatiotemporal parameters at a common speed for a wide range of endurance runners (i.e., 12 km·h^−1^). In total, 97 runners, 52 men and 45 women, completed a treadmill running protocol at 12 km·h^−1^. Spatiotemporal parameters were measured using the OptoGait system, and foot structure was assessed by determining arch stiffness. Since between-sex differences were found in anthropometric and foot structure variables, data analysis was conducted separately for men and women, and body mass and height were considered as covariates. For both sexes, a k-means cluster analysis grouped participants according to arch stiffness, by obtaining a group of low-arch stiffness (LAS group) and a group of high-arch stiffness (HAS group), with significant differences in arch stiffness (*p* < 0.001, for both men and women). No significant differences between LAS and HAS groups were found in running spatiotemporal parameters, regardless of sex (*p* ≥ 0.05). For both sexes, the partial correlation analysis reported no significant correlations (*p* ≥ 0.05) between foot structure variables and running spatiotemporal parameters. The results obtained show no differences in spatiotemporal gait characteristics during running at submaximal velocity between runners with low-arch stiffness and those with high-arch stiffness, regardless of sex. These findings may have important implications for clinicians and coaches by adding more evidence to the debate about the use of static foot classification measures when characterizing the foot and its biomechanics during running.

## 1. Introduction

Considering that the foot interacts with the ground during gait, any change here might imply compensatory malalignment, not only in the lower extremities, but also in the entire body [1,2,3,4]. In fact, some static foot measures have been considered a risk factor for running-related injuries and an influencing factor for overuse injuries in runners [2,3]. Nevertheless, this is a controversial topic. The premise that structure dictates function underpins the use of static foot measurements to predict and explain the functional capacity of the foot in action [5]. However, the relationship between structure and function in terms of static foot posture and dynamic foot motion is not clearly understood [2,6].

Differences in lower extremity kinematics and kinetics between high- and low-arched runners have already been studied [1,7,8], reporting arch mobility as independent of the static height of the medial longitudinal arch [9,10]. It has been suggested that reduced arch mobility relates to a growing need to comply with other lower extremity joints (i.e., knee) [7]. However, a flexible arch or a midfoot that compresses under force may result in increased pronation of both midfoot and rearfoot [8]. As a result of this shift in rearfoot pronation, it has been suggested that a runner with a mobile high arch might show different lower extremity kinematics and kinetics in comparison with a runner with low-arch mobility [8].

Nevertheless, limited evidence is available on arch stiffness and its role within the spring-mass model during running [5,6,7,10]. Stiffness has been proposed as the key mechanical parameter for the spring-mass model [11,12]. It represents the capacity of the lower extremity as a whole to alleviate the excessive forces produced during the running stance phase [13,14]. When decreased stiffness is present, the system becomes too compliant, which might cause both overuse and overload of the structures involved in the force attenuation phase. In contrast, higher levels of stiffness (decreased compliance) may cause an increase in forces up the kinetic chain [12,13,15]. Lower-body stiffness during running has been extensively associated with spatiotemporal parameters [11,16,17]. Among spatiotemporal gait characteristics, the ground contact time (CT) seems to be the major determinant of stiffness during running. Morin and colleagues [17] developed a simple method for estimating leg stiffness from a few mechanical and anthropometrical parameters and determined, through a sensitive analysis, that ground CT showed a higher relative influence on the calculated stiffness than the rest of the parameters constituting the model.

To the best of the authors’ knowledge, no previous studies have determined the influence of arch stiffness on spatiotemporal parameters during running. However, it is well known that the structure of the foot enables it to serve as a shock attenuator in the early and midstance and as a rigid lever during toes-off, making the arch principally responsible for such functions [7,18]. Therefore, taking the above information into consideration, it makes sense to affirm that arch stiffness could be a determining factor of the leg-spring behavior during running.

Earlier works [4,5,7,8,9,10] investigated the similarities between static foot measures and dynamic foot movements while running, but none considered arch stiffness and its effect on running biomechanics. Therefore, the aim of this study is to determine the influence of arch stiffness on running spatiotemporal parameters at a comfortable speed in endurance runners. The authors hypothesized that runners with stiffer arches would exhibit different lower extremity kinematics during running compared to those with compliant arches.

## 2. Materials and Methods

### 2.1. Participants

A total of 52 men and 45 women (*n* = 97) (age: 27 ± 8 years; range: 18–40 years; height: 172 ± 9 cm; body mass: 66 ± 10 kg; experience in running: 3.7 ± 1.5 years; weekly mileage: 36 ± 11 km) voluntarily participated in this study. All participants met the inclusion criteria: (i) over 18 years old; (ii) recreationally trained endurance runners (3–4 running sessions per week, at least once on a treadmill); (iii) able to run 5 km in less than 25 min; (iv) have not suffered from any injury within the last 6 months before data collection. The sample size was selected by convenience. Nevertheless, a post-hoc analysis of the achieved power for this sample size was conducted (G*Power software vs. 3.1) [19], and it revealed a high power (>0.8). This study was conducted in accordance with the latest version of the World Medical Association’s Declaration of Helsinki [20]. Each participant received detailed information on the procedures and signed an informed consent form. Additionally, the study was approved by the institutional review board (Ethical Committee of the San Jorge University, code 009-17/18).

### 2.2. Design and Procedures

Participants were individually tested in one day. Prior to all testing, participants refrained from severe physical activity for at least 48 h, and all testing was conducted at least 3 h after ingesting a meal.

A standardized 10-min accommodation warm-up preceded the motorized treadmill protocol (Salter M-835, Salter Int., Barcelona, Spain). Athletes were familiar with treadmill running, although previous works on human locomotion have shown that accommodation to a new condition occurs in 6–8 min [21,22]. The accommodation period was developed at 10 km·h^−1^. After that, running velocity increased 1 km·h^−1^ every minute until the velocity of 12 km·h^−1^ was reached. Then, participants maintained that velocity for 1 min with an acclimatization period of 45–50 s and a recording period of 10 s [23]. This pace has been shown to be sufficient for the participants, and it is in line with previous studies [24]. At the set velocity, all participants reported feeling comfortable while running on the treadmill. The short duration of the periods (i.e., 1 min) helped to minimize the impact of fatigue on running kinematics.

### 2.3. Measures

(i) For descriptive purposes, both body height (cm) and mass (kg) were measured, employing a stadiometer and weighing scale (SECA 222 and 634, respectively, SECA Corp., Hamburg, Germany).
(ii) The OptoGait system (Optogait; Microgate, Bolzano, Italy) was used to calculate the spatiotemporal parameters, which has been previously validated for the assessment of gait characteristics (i.e., spatiotemporal parameters), exhibiting a good validity and reliability [25]. The system, composed of two parallel bars, was placed on both edges of the treadmill leveled with the contact surface. The device was linked to a computer where data were recorded. Limb dominance was not considered [26]. For every step, the next variables were obtained:
  - Contact time (CT, in seconds): period of time from the earliest contact of the foot with ground to toes-off.
  - Flight time (FT, in seconds): span of time from toes-off to initial collision with ground contact of the consecutive foot (i.e., right–left).
  - Step length (SL, in meters): length the treadmill belt advances from toes-off to initial ground contact in successive steps.
  - Step frequency (SF, in steps/min): number of ground contacts per minute.
  - Step angle (°): angle of the parable tangent derived from SL and the height calculated from FT. These parameters enable tying in SL with FT and defining it as bound [27]. The maximal height for a stride is calculated by the OptoGait system as:
    *h = (g × FT^2^)/8* (1)
    where *h* means height and *g* means gravity (*g* = 9.81 m.s^−2^).
  - The percentage of ground CT at which the different subphases of stance occur (based on active LEDs): initial contact (Phase 1: time from the earliest ground contact to flat foot), midstance (Phase 2: time from flat foot to initial take off), and propulsion (Phase 3: time from initial take off to toes-off). This was also automatically measured for every step during the treadmill test by the OptoGait system.
(iii) Foot assessments were developed by the same experienced researcher. The mean value of three repeated measurements was registered and employed for subsequent analysis. The static foot posture and foot mobility measurements have shown moderate to good intra-rater reliability (ICC 0.81–0.99) and moderate to good inter-rater reliability (ICC 0.58–0.99) [28,29].

Both arch height and stiffness were used to characterize foot structure. Arch height was defined as the height of the dorsum of the foot normalized to truncated foot length [7]. Arch stiffness was defined as the change in the arch height index (AHI) due to the increase in load between sitting and standing conditions [30]. One single researcher took these measurements using the AHI Measurement System [30]. Butler et al. [30] reported high intra-rater and inter-rater reliability. Participants were asked to sit in a height-adjustable chair. The chair was then adjusted to keep knees and hips under a 90° alignment and with slight contact between the plantar foot surface and the measurement platform. A specially designed platform for undertaking this measurement was used [28]. A digital caliper was used to measure the dorsum of the foot at 50% of total foot length. Then, from the most posterior aspect of the calcaneus, which was fixed at a heel cup, to the most distal aspect of the longest toe, the total foot length was considered. It was repeated in a bipedal stance position assuming body weight. Both feet were fixed in the heel cups positioned 15 cm apart. The dorsal arch height difference was calculated as the difference between dorsal arch in bipedal standing and in sitting position, known as the sit-to-stand difference [31], whereas the AHI was calculated as [29]:

*AHI = Dorsum Height/Truncated Foot Length* (2)

Based on previous studies [18], arch stiffness was calculated assuming a 40% change in load between sitting and standing conditions:

*Arch Stiffness = (0.40 × BodyMass)/(AHI (seated) − AHI (standing))* (3)

### 2.4. Statistical Analysis

Descriptive statistics were represented as the mean and standard deviation (SD). Normal distribution and homogeneity were confirmed through Shapiro–Wilk and Levene’s test, respectively. An analysis of covariance (ANCOVA, with body height and body mass as covariates) was conducted to examine the differences between sexes in foot--related variables and spatiotemporal parameters. Since between-sex differences have been reported in arch stiffness [18], data analysis was conducted separately for men and women. For each sex, a k-means cluster analysis was performed by grouping according to the arch stiffness (low-arch stiffness group (LAS) and high-arch stiffness group (HAS)). Those subgroups (LAS vs. HAS) were compared by means of an ANCOVA (with body height and body mass as covariates). Additionally, a partial correlation analysis adjusted by body height and body mass was performed for each sex between the variables—foot structure, mobility, and spatiotemporal parameters. Statistical analysis was conducted using SPSS software (version 21, SPSS Inc., Chicago, IL, USA). The level of significance was set at *p* < 0.05.

## 3. Results

Between-sex differences were found in anthropometric variables and foot structure variables, but no differences were found in spatiotemporal gait characteristics during running (Table 1). Men were taller (*p* < 0.001) and heavier (*p* < 0.001) than women. Additionally, men showed greater arch stiffness (*p* = 0.008), and unweighted and weighted dorsal arch height (*p* < 0.05).

The k-means cluster analysis grouped men into a LAS group (*n* = 41, arch stiffness = 759.55 body mass/AHI units) and a HAS group (*n* = 11, arch stiffness = 1729.69 body mass/AHI units) and grouped women into a LAS group (*n* = 37, arch stiffness = 698.04 body mass/AHI units) and a HAS group (*n* = 8, arch stiffness = 1785.49 body mass/AHI units).

Significant differences between groups (LAS vs. HAS) were found in anthropometric variables in men (*p* < 0.05), with no between-group differences in women (*p* ≥ 0.05). For men, the HAS group showed greater values in body height (*p* = 0.001) and body mass (*p* = 0.05) than the LAS group (Table 2).

Significant differences between HAS and LAS groups were found in the variables of foot structure and mobility (Table 3). In both sexes, the HAS group showed greater arch stiffness (*p* < 0.001), smaller dorsal arch height difference (*p* = 0.001), and smaller AHI difference (*p* < 0.001).

No significant differences between runners with lower-arch stiffness (LAS group) and those with higher-arch stiffness (HAS group) were found in spatiotemporal gait characteristics during running at 12 km·h^−1^ (Table 4). No between-group differences were found in either men or in women (*p* ≥ 0.05).

For both sexes, the partial correlation analysis, adjusted by body height and body mass, reported no significant correlations (*p* ≥ 0.05) between the variables of foot structure and mobility (arch stiffness, dorsal arch height difference, and AHI difference) and spatiotemporal parameters during running.

## 4. Discussion and Conclusions

The purpose of this study was to examine whether arch stiffness influences spatiotemporal parameters while running at a comfortable speed (12 km·h^−1^). The main finding of the current study is the lack of differences in running spatiotemporal characteristics between runners with lower-arch stiffness and those with high-arch stiffness, regardless of sex. To note, the partial correlation analysis performed in this work reinforces such findings by exhibiting no relationships between foot structure variables and running spatiotemporal parameters at submaximal velocities. This finding may have important implications for clinicians and coaches by adding more evidence to the debate about the use of static foot classification measures when characterizing the foot and its biomechanics during running. Additional findings of this study are: (i) the presence of between-sex differences in foot structure and mobility (i.e., men showed greater arch stiffness than women) and (ii) the lack of relationship between static arch measures (i.e., dorsal arch height) and arch deformation (i.e., dorsal arch height deformation and arch stiffness).

In order to interpret the results properly, three points should be considered: (i) as between-sex differences had been reported in arch stiffness [18] and the current work reinforces that finding, data analysis was conducted separately for men and women; (ii) despite the fact that statistical analysis was conducted separately for men and women, some differences between LAS and HAS groups were found in anthropometric characteristics and, therefore, body mass and height were considered covariates for the subsequent analysis; and (iii) the completed treadmill protocol aimed to avoid the influence of fatigue on running biomechanics [26].

As mentioned above, previous studies have focused on arch height influences rather than how the arch works when standing (e.g., arch stiffness). For instance, Williams et al. [4] compared low- and high-arched runners and found a significant relationship between arch structure (i.e., arch height) and lower extremity kinematics and kinetics during running. In an attempt to avoid the potential influence of arch height and to specifically address the influence of the amount of mobility in the medial longitudinal arch, Williams et al. [8] focused on high-arched runners with a rearfoot strike pattern to determine the influence of arch mobility on running kinematics and ground reaction forces. The authors [8] found that arch mobility in high-arched runners had a significant effect on tibial-rotation excursion and ground reaction forces. The findings from both studies suggest that the assessment of static arch mobility might be an important factor in understanding the lower extremity function during running. Nevertheless, the evidence about the association between structure and function is not clearly understood and remains inconclusive [2,6]. Langley et al. [6] aimed to determine whether the static foot measures (i.e., foot posture index, medial longitudinal arch and rearfoot angle in a standing position) predicted medial longitudinal arch deformation during running barefoot at a comfortable speed, but no significant relationship between those variables was reported. Similar results were found by Lees et al. [9], who suggested that the arch height does not seem to play an important role in dynamic foot motion. The lack of methodological consensus (different populations, running velocity, and outcome measures) makes the comparison difficult, but in this controversial context, the current study provides some insights into the relationship between arch stiffness and running spatiotemporal parameters considering sex and adjusting by body mass and height.

Another interesting finding of the current study is the presence of between-sex differences in arch stiffness. Specifically, men showed greater arch stiffness than women, and smaller dorsal arch height in both weighted and unweighted conditions. These data provide support to a previous study [18] that reported differences in arch deformation between sexes, with men showing greater arch stiffness than women.

Concerning the lack of differences in dorsal arch height between LAS and HAS groups, the results obtained are in line with those reported by a previous study [6].

The authors found that static foot measures do not predict arch deformation during dynamic tasks and thus questioned the objective of using static foot classification measurements when characterizing the foot during running. Likewise, Nachbauer and Nigg [10] found no association between static and dynamic foot measures, specifically between static medial longitudinal arch height and its deformation during running.

Finally, some limitations must be taken into consideration. First, the effect of running velocity and the kinematic adaptations to increased velocity were not considered. It is possible that kinematic adaptations at higher running velocities are related to arch mobility. Second, further differences between groups may be revealed in a deeper biomechanical analysis (joint angles and kinetic variables). Third, the foot-strike pattern exhibited by runners was not controlled. Differences exist in running biomechanics for forefoot, midfoot, and rearfoot strikers [32]. Consequently, different responses to foot structure and mobility might be found. Notwithstanding these limitations, the current paper highlights the lack of differences in running spatiotemporal parameters between runners with lower-arch stiffness and those with high-arch stiffness in a sample of 97 endurance runners.

The results obtained in this study showed no differences in spatiotemporal gait characteristics during running at submaximal velocity (12 km·h^−1^) between runners with low-arch stiffness and those with high-arch stiffness, regardless of sex. From a practical standpoint, the lack of a relationship between foot structure variables and running spatiotemporal parameters has important implications for clinicians and coaches by adding more evidence to the debate about the use of static foot classification measures when characterizing the foot and its biomechanics during running. In this context, further research is clearly needed to determine the potential relationship between other structures (e.g., tendons or fascia) and its biomechanics during dynamic actions such as running or jumping.

## Figures and Tables

**Table 1:** Sex comparison in outcome variables (anthropometric, foot structure, and spatiotemporal parameters)**.**

Variable	Total (*n* = 97)	Men (*n* = 52)	Women (*n* = 45)	*p*-Value
Body height (cm) †	172.02 (8.97)	177.71 (7.27)	165.44 (5.61)	<0.001
Body mass (kg) †	66.00 (10.27)	72.94 (7.93)	57.98 (5.87)	<0.001
Age (years) †	25.66 (7.54)	27.73 (7.56)	25.42 (7.41)	0.134
Foot structure variables				
Arch stiffness (body mass/AHI units)	930.72 (468.26)	964.77 (460.10)	891.36 (479.66)	0.008
Unweighted DAH (cm)	6.52 (0.53)	6.77 (0.42)	6.24 (0.51)	0.024
Weighted DAH (cm)	6.00 (0.44)	6.22 (0.39)	5.74 (0.40)	0.034
DAH difference (cm)	0.53 (0.30)	0.55 (0.23)	0.50 (0.37)	0.339
Unweighted AHI	0.36 (0.03)	0.35 (0.02)	0.36 (0.03)	0.271
Weighted AHI	0.32 (0.02)	0.32 (0.02)	0.33 (0.03)	0.854
AHI difference	0.04 (0.02)	0.04 (0.01)	0.03 (0.02)	0.135
Spatiotemporal gait characteristics				
FT (s)	0.071 (0.019)	0.069 (0.020)	0.073 (0.019)	0.679
CT (s)	0.292 (0.020)	0.296 (0.022)	0.288 (0.017)	0.062
SL (cm)	120.89 (6.18)	121.49 (6.12)	120.20 (6.25)	0.200
SF (steps/min)	165.87 (8.36)	165.08 (8.35)	166.79 (8.37)	0.128
Step angle (°)	1.26 (0.66)	1.20 (0.66)	1.34 (0.67)	0.478
Phase 1 (%)	13.04 (2.69)	13.18 (2.88)	12.89 (2.47)	0.641
Phase 2 (%)	51.90 (3.67)	50.66 (3.36)	53.33 (3.52)	0.473
Phase 3 (%)	35.06 (3.09)	36.16 (2.88)	33.78 (2.85)	0.199

† indicates that no covariates were considered to compare sexes; DAH: dorsal arch height; DAH difference: dorsal arch height difference (unweighted–weighted); LAS: low-arch stiffness group; HAS: high-arch stiffness group; AHI: arch height index; FT: flight time; CT: contact time; SL: step length; SF: step frequency; Phase 1, 2 and 3, are different subphases during ground contact: Phase 1 (initial contact), Phase 2 (midstance), and Phase 3 (propulsion).

**Table 2:** Characteristics of participants according to the created subgroups (LAS vs. HAS groups), for both men and women.

**Men (** ***n*** ** = 52)**				
**Variable**	**Total** (***n***** = 52)**	**LAS Group** (***n***** = 41)**	**HAS Group** (***n***** = 11)**	***p*** **-Value**
Body height (cm) †	177.71 (7.27)	176.02 (6.43)	184.00 (7.01)	0.001
Body mass (kg) †	72.94 (7.93)	71.34 (7.74)	78.77 (5.84)	0.005
Age (years) †	27.73 (7.56)	28.72 (8.05)	24.01 (3.56)	0.066
**Women *(n = 45)***				
**Variable**	**Total** **(*n* = 45)**	**LAS group** **(*n* = 37)**	**HAS group** **(*n* = 8)**	***p*-value**
Body height (cm) †	165.44 (5.61)	165.73 (5.76)	168.75 (3.45)	0.065
Body mass (kg) †	57.98 (5.87)	57.24 (5.36)	61.38 (7.31)	0.071
Age (years) †	25.42 (7.41)	26.01 (8.05)	22.73 (1.34)	0.262

† indicates that no covariates were considered to compare groups; LAS: low-arch stiffness group; HAS: high-arch stiffness group.

**Table 3:** Foot structure variables according to the groups’ comparison, for both men and women.

**Men (** ***n*** ** = 52)**				
**Variable**	**Total** (***n***** = 52)**	**LAS Group** (***n***** = 41)**	**HAS Group** (***n***** = 11)**	***p*** **-Value**
Arch stiffness (body mass/AHI units)	964.77 (460.10)	759.55 (196.27)	1729.69 (330.56)	<0.001
Unweighted DAH (cm)	6.77 (0.42)	6.80 (0.39)	6.64 (0.53)	0.086
Weighted DAH (cm)	6.22 (0.39)	6.19 (0.35)	6.32 (0.53)	0.929
DAH difference (cm)	0.55 (0.23)	0.62 (0.22)	0.33 (0.10)	0.001
Unweighted AHI	0.35 (0.02)	0.36 (0.02)	0.34 (0.03)	0.240
Weighted AHI	0.32 (0.02)	0.32 (0.02)	0.32 (0.03)	0.269
AHI difference	0.04 (0.01)	0.04 (0.01)	0.02 (0.00)	<0.001
**Women (*n* = 45)**				
**Variable**	**Total** **(*n* = 45)**	**LAS Group** **(*n* = 37)**	**HAS Group** **(*n* = 8)**	***p*-Value**
Arch stiffness (body mass/AHI units)	891.36 (479.66)	698.04 (213.40)	1785.49 (317.43)	<0.001
Unweighted DAH (cm)	6.24 (0.51)	6.27 (0.55)	6.11 (0.25)	0.105
Weighted DAH (cm)	5.74 (0.40)	5.71 (0.37)	5.89 (0.28)	0.552
DAH difference (cm)	0.50 (0.37)	0.55 (0.38)	0.22 (0.11)	0.005
Unweighted AHI	0.36 (0.03)	0.36 (0.03)	0.33 (0.03)	0.019
Weighted AHI	0.33 (0.03)	0.33 (0.02)	0.32 (0.03)	0.784
AHI difference	0.03 (0.02)	0.04 (0.02)	0.01 (0.02)	0.001

LAS: low-arch stiffness group; HAS: high-arch stiffness group; DAH difference: dorsal arch height difference (unweighted–weighted); AHI: arch height index.

**Table 4:** Spatiotemporal variables during running at 12 km·h^−1^ according to the created subgroups (LAS vs. HAS), for both men and women.

**Men (** ***n*** ** = 52)**				
**Variable**	**Total** (***n***** = 52)**	**LAS Group** (***n***** = 41)**	**HAS Group** (***n***** = 11)**	***p*** **-Value**
FT (s)	0.069 (0.020)	0.070 (0.021)	0.065 (0.014)	0.898
CT (s)	0.296 (0.022)	0.293 (0.024)	0.306 (0.012)	0.953
SL (cm)	121.49 (6.12)	121.02 (6.16)	123.19 (5.93)	0.842
SF (steps/min)	165.08 (8.35)	165.75 (8.52)	162.58 (7.56)	0.860
Step angle (°)	1.20 (0.66)	1.26 (0.71)	1.01 (0.36)	0.966
Phase 1 (%)	13.18 (2.88)	13.26 (2.86)	12.88 (3.09)	0.353
Phase 2 (%)	50.66 (3.36)	50.44 (3.44)	51.47 (3.06)	0.063
Phase 3 (%)	36.16 (2.88)	36.30 (2.91)	35.65 (2.82)	0.126
**Women (*n* = 45)**				
**Variable**	**Total** **(*n* = 45)**	**LAS Group** **(*n* = 37)**	**HAS Group** **(*n* = 8)**	***p*-Value**
FT (s)	0.073 (0.019)	0.073 (0.020)	0.072 (0.016)	0.923
CT (s)	0.288 (0.017)	0.287 (0.018)	0.292 (0.013)	0.982
SL (cm)	120.20 (6.25)	120.00 (6.20)	121.10 (6.86)	0.978
SF (steps/min)	166.79 (8.37)	167.05 (8.37)	165.58 (8.86)	0.913
Step angle (°)	1.34 (0.67)	1.35 (0.70)	1.25 (0.53)	0.806
Phase 1 (%)	12.89 (2.47)	13.27 (2.30)	11.14 (2.62)	0.059
Phase 2 (%)	53.33 (3.52)	53.31 (3.34)	53.43 (4.51)	0.768
Phase 3 (%)	33.78 (2.85)	33.43 (2.56)	35.43 (3.64)	0.131

LAS: low-arch stiffness group; HAS: high-arch stiffness group; FT: flight time; CT: contact time; SL: step length; SF: step frequency; Phase 1, 2 and 3, are different subphases during ground contact: Phase 1 (initial contact), Phase 2 (midstance), Phase 3 (propulsion).

## Data Availability

Data will be available by request.
